# Three-Dimensional Assessment of Bilateral Symmetry of the Scaphoid: An Anatomic Study

**DOI:** 10.1155/2015/547250

**Published:** 2015-08-27

**Authors:** Paul W. L. ten Berg, Johannes G. G. Dobbe, Simon D. Strackee, Geert J. Streekstra

**Affiliations:** ^1^Department of Plastic, Reconstructive, and Hand Surgery, Academic Medical Center, University of Amsterdam, Meibergdreef 9, 1105 AZ Amsterdam, Netherlands; ^2^Department of Biomedical Engineering and Physics, Academic Medical Center, University of Amsterdam, Meibergdreef 9, 1105 AZ Amsterdam, Netherlands; ^3^Department of Radiology, Academic Medical Center, University of Amsterdam, Meibergdreef 9, 1105 AZ Amsterdam, Netherlands

## Abstract

Preoperative 3D CT imaging techniques provide displacement analysis of the distal scaphoid fragment in 3D space, using the matched opposite scaphoid as reference. Its accuracy depends on the presence of anatomical bilateral symmetry, which has not been investigated yet using similar techniques. Our purpose was to investigate symmetry by comparing the relative positions of distal and proximal poles between sides. We used bilateral CT scans of 19 adult healthy volunteers to obtain 3D scaphoid models. Left proximal and distal poles were matched to corresponding mirrored right sides. The left-to-right positional differences between poles were quantified in terms of three translational and three rotational parameters. The mean (SD) of ulnar, dorsal, and distal translational differences of distal poles relative to proximal poles was 0.1 (0.6); 0.4 (1.2); 0.2 (0.6) mm and that of palmar rotation, ulnar deviation, and pronation differences was −1.1 (4.9); −1.5 (3.3); 1.0 (3.7)°, respectively. These differences did not significantly differ from zero and thus were not biased to left or right side. We proved that, on average, the articular surfaces of scaphoid poles were symmetrically aligned in 3D space. This suggests that the contralateral scaphoid can serve as reference in corrective surgery. No level of evidence is available.

## 1. Introduction

A scaphoid waist fracture with displacement in which the proximal and distal poles are malaligned is seen as an indication for surgery [1]. Scaphoid fractures that failed to unite (i.e., nonunions) are associated with a flexion deformity with bone loss around fracture sites, in which the distal fragment rotates in palmar direction. Besides achieving union, the surgeon's goal is to adequately restore the normal scaphoid alignment by an adequate reduction of the fragments. In the treatment of scaphoid nonunions, the surgeon often uses an interpositional cortical bone graft between the fragments to accomplish this goal [1]. Failure of restoring alignment results in a malunited scaphoid which may lead to pain and restricted motion [2].

In current clinical practice, assessment of displacement is based on measurements using two-dimensional (2D) images (i.e., radiographs and single CT slices) which is subjective due to manual measurements, position of the wrist during imaging, and/or slice selection [3, 4]. This method of assessment has shown to be poorly reliable and is prone to inter- and intraobserver variability [5–7], making clinical decision making and surgical planning difficult. For an optimal surgical planning, a 3D approach is required to adequately restore the anatomical alignment of the articular surfaces of proximal and distal poles, since fracture displacement is a 3D problem [2].

**Figure 1:**
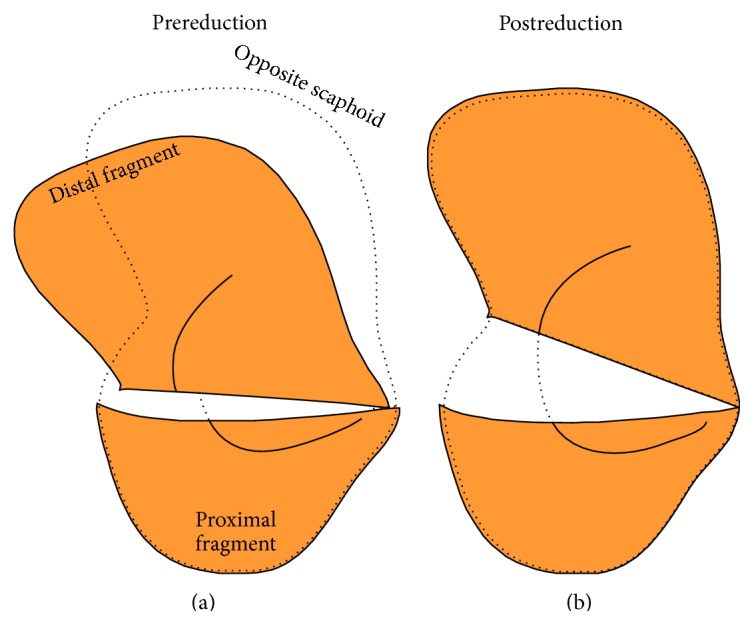
Scheme of 3D model of scaphoid fragments before virtual reduction (a) and after reduction (b). The mirrored opposite scaphoid (dotted outline) serves as guide to virtually reduce the nonunion fragments. This method enables quantifying the amount of displacement of the distal fragment in 3D space.

Quantitative 3D CT imaging techniques can be applied to assess the level of scaphoid fracture displacement in 3D space, demonstrated by several recently published studies [2, 8, 9]. This technique is independent of imaging position of the wrist or slice selection. It is based on virtual reduction of the proximal and distal fragments, using the position of the proximal and distal poles of the opposite mirrored healthy scaphoid as template (Figure 1). After virtual reduction, the amount of displacement of the distal scaphoid fragment can be quantified in terms of three translational parameters (ulnar, dorsal, and distal) and three rotational parameters (palmar flexion, ulnar flexion, and pronation).

A prerequisite for a reduction technique that uses the opposite scaphoid as reference is the presence of normal bilateral symmetry [10]. Asymmetry may be a limiting factor in the accuracy of this displacement analysis. Using such a potentially biased displacement analysis for surgical planning may cause an inconsistency between the achieved postreduction position and desired pretraumatic position of the distal scaphoid fragment. However, there are no reports quantifying symmetry in terms of the translational and rotational differences of scaphoid poles in 3D space. Therefore, it is unclear whether or not results of the level of fracture displacement are systematically biased in existing or future clinical scaphoid studies relying on these imaging techniques.

The purpose of this anatomic 3D CT study was to investigate the symmetry of healthy scaphoid pairs. To this end, we quantified side-to-side differences of the positions of distal poles within healthy scaphoid pairs in terms of the three translational and three rotational parameters. We hypothesize that there is no bias to the left or right side in each of these parameters, showing average difference values not significantly different from zero.

## 2. Materials and Methods

### 2.1. Data Acquisition

Nineteen healthy right-handed volunteers participated in this study (13 women and six men; average age: 26 y; range: 22–56 y). The subjects had no history of wrist injury or other musculoskeletal disorders. A high-resolution CT scan (Philips Brilliance 64 CT scanner, Cleveland, OH) was made of both wrists (i.e., bilateral CT scan) of each individual using standardized methods (voxel size 0.45  ×  0.45  ×  0.45 mm., 120 kV, 150 mAs, pitch 0.6, and slice thickness 0.67 mm.). The CT scans were used for subsequent 3D image analyses. To determine the methodological accuracy and reproducibility of our method, one cadaver arm was scanned multiple times (10x), using the same scan protocol. This study was approved by our Human Research Committee. Informed consent of each individual was obtained prior to participation.

### 2.2. Assessment of Side-to-Side Positional Differences

**Figure 2:**
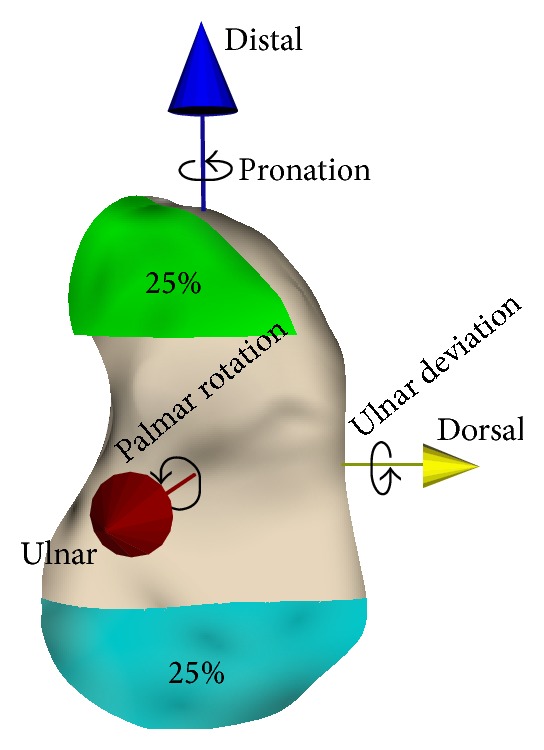
Virtual model of a left scaphoid with anatomical coordinate system, defining translational and rotational differences. After matching the proximal (blue; 25%) poles, side-to-side differences are shown as the degree in which the positions of the distal poles (green; 25%) differ between the left and right sides.

First, from each scaphoid pair, the left scaphoid is segmented from a CT scan, based on custom made software [10]. A 3D polygon mesh from the segmented data is derived which served as a virtual 3D model of the bone. To allow comparison of side-to-side differences between subjects in an unambiguous fashion, an anatomical coordinate system is defined for every scaphoid model based on its inertial properties (Figure 2) [11].

Next, we selected a proximal and a distal pole of 25% of the total length of the left scaphoid (Figure 2). The central 50% of the scaphoid, in which the fracture usually occurs [12], is therefore omitted from this analysis. Next, the left scaphoid is matched with the mirrored CT image of the right scaphoid by aligning the proximal poles using intensity-based image registration [13].

For this registration process, first, a 3D double-contour polygon is automatically created based on the initial 3D polygon mesh of the left scaphoid by sampling the image intensity 0.3 mm toward the inside (high CT value) and outside (low CT value) of the bone, along the surface normal vector. The points of the double-contour polygon of the left proximal pole are registered with the reference image of the mirrored right scaphoid in a rigid point-to-image registration procedure [13]. This procedure uses the Nelder-Mead downhill simplex optimizer with a six-parameter search space (three displacements and three rotations) while the correlation coefficient was used as metric unit, which quantifies how well the gray-level points fit the reference image [14, 15]. The use of a double-contour polygon makes the registration highly discriminative [13].

Then, side-to-side differences are expressed as the degree in which the positions of the distal poles differ, relative to the proximal pole, between left and right scaphoids. The three translational differences (ulnar, dorsal, and distal translational) and three rotational differences (palmar rotation, ulnar deviation, and pronation) are derived from the 4 × 4 transformation matrices that resulted from image registration [13].

Statistical analyses of the measurements included the Shapiro Wilks W test as normality test, determining the mean and standard deviation (SD) for normally distributed data. A one-sample *t*-test was used to investigate whether the six means of the translational and rotational differences differ significantly from zero. A post hoc power analysis for one-sample *t*-test was used to calculate the level of mean side-to-side differences that could have been tested on significance with sufficient power. This power analysis requires input of the sample size (*N* = 19), comparison mean (=0) and standard deviation, while using an *α*-level of 0.05 and a power of 0.80. A 5% significance level was used for the analyses.

### 2.3. Accuracy and Reproducibility of the Method

**Figure 3:**
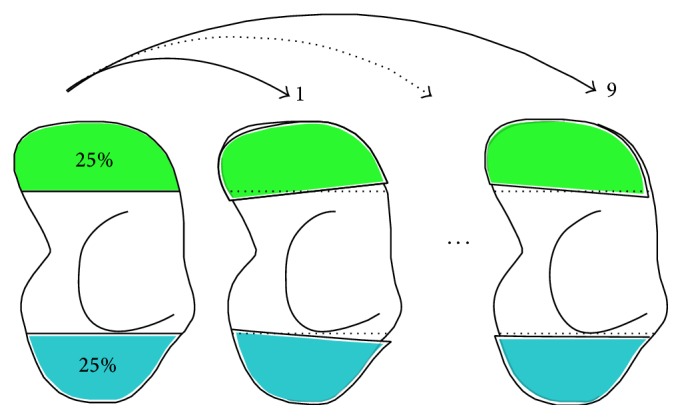
Scheme of the cadaveric experiment assessing the accuracy and reproducibility of the matching procedure. After scanning one cadaveric arm tenfold, the scaphoid from one CT scan was segmented (left model). The proximal (blue) poles were matched to the remaining 9 scans enabling displacement analysis of the distal (green) pole.

We assessed the accuracy and reproducibility of our method by investigating the influence of the segmentation and matching procedure on translational and rotational side-to-side differences of the distal poles. To this end, we used ten CT scans of a single cadaveric arm. For each scan, the arm was scanned at a slightly different position inside the scanner to include possible variations in the reconstructed 3D image due to different positions of the wrist. Hereafter, a single 3D model of the scaphoid was obtained from the first CT scan. The proximal pole of this scaphoid model was selected and subsequently matched to the remaining nine scans of the same cadaver arm (Figure 3). The change in relative position of the distal pole with respect to the proximal pole before and after matching yields the “side-to-side positional differences” due to methodological errors. A zero mean of these differences indicates a high level of methodological accuracy, because, in this experimental study, true symmetry is present since the scaphoid is matched to itself. The standard deviation represents the reproducibility of the method.

## 3. Results

### 3.1. Accuracy and Reproducibility

Accuracy and reproducibility of radioulnar, palmodorsal, and proximodistal translation of the distal pole relative to the proximal pole were (mean (SD)) −0.1 (0.1), 0.1 (0.1), and 0.0 (0.1) mm, respectively. Accuracy and reproducibility of dorsopalmar rotation, radioulnar deviation, and supination and pronation deviation of the distal pole relative to the proximal pole were (mean (SD)) equal to −0.1 (0.5), 0.1 (0.4), and −0.1 (0.3) degrees, respectively. All means did not deviate more than a tenth of a millimeter or degree from zero indicating a high level of methodological accuracy. All standard deviations were lower than a tenth of a millimeter or half a degree, indicating a relatively high reproducibility.

### 3.2. Side-to-Side Positional Differences

**Figure 4:**
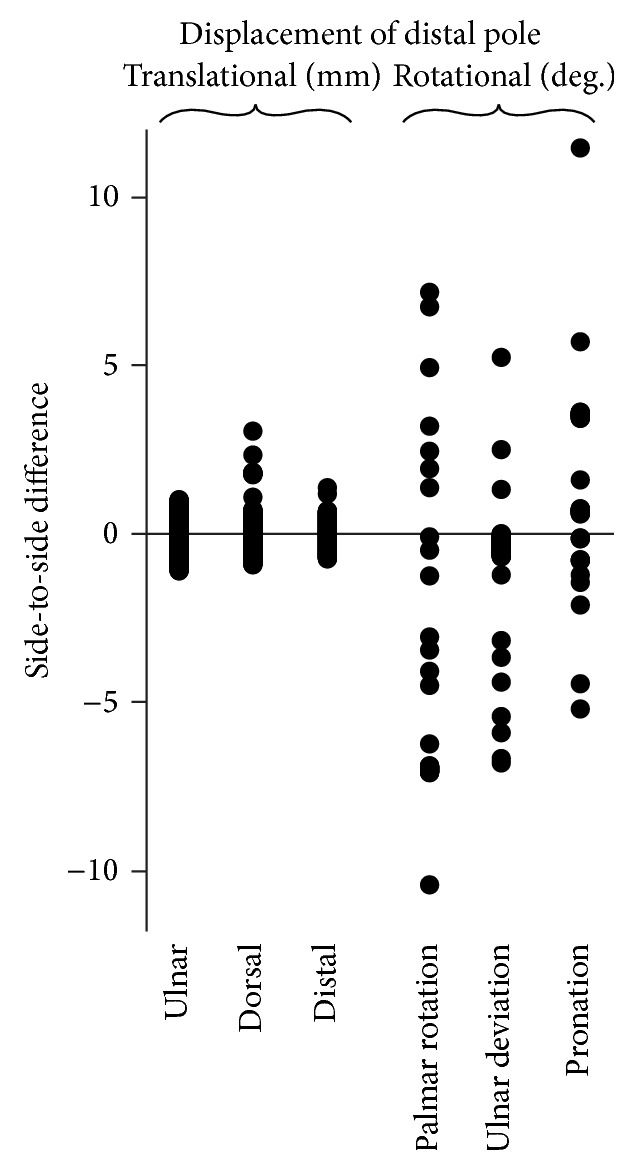
Scatterplot showing the left-to-right alignment differences of the distal poles of the 19 uninjured scaphoid pairs. Each dot represents a side-to-side difference for an individual healthy subject expressed in terms of an anatomical coordinate system (Figure 2). Negative displacement values represent opposite directions.

Values of all translational and rotational differences of the distal poles of the 19 scaphoid pairs were normally distributed. Corresponding means and standard deviations are listed in Table 1. To perform the power analysis, we calculated the average standard deviation (SD) of the 3 translational differences (mm) and of the 3 rotational differences (°), resulting in a SD of ±0.8 mm and SD of ±4.0°. Based on these SDs, there was sufficient power to detect a significant left-right bias if mean side-to-side differences were >0.5 mm, regarding translational differences, and a significant left-right bias if mean side-to-side differences were >2.6°, regarding rotational differences. Our reported mean side-to-side differences were smaller than these cut-offs and were not statistically different from zero (Table 1 and Figure 4). This indicated that, in our sample, differences were not biased to a left or right side. The spread in these differences due to individual left-to-right variability is much larger than the methodological errors found above.

## 4. Discussion

We used a quantitative 3D CT method to investigate the degree of positional differences of distal poles between healthy scaphoids sides. The proposed method of evaluation includes determination of an anatomical coordinate system that permits objectively comparing side-to-side differences of different individuals. The applied technique has proven to be accurate and highly reproducible. Overall, the translation and rotation differences between sides did not significantly differ from zero. This implied that there was no bias to the left or right side, indicating anatomical bilateral symmetry of the scaphoid poles in 3D space.

A limitation of our study is that all participants were right handed, which does not provide information about the side-to-side differences in left-handed individuals. Despite being not proven in this study, we expect similar results for left-handed individuals.

In upper extremity surgery, the opposite healthy bone can be used to plan and guide reconstruction of complex fractures [16, 17]. However, the presence of bilateral asymmetry in bone shape could hamper using the opposite bone as a reference for planning. Studies have documented greater right biases in upper limb bone dimensions especially in length [18, 19]. For example, in healthy radius pairs, there is a length bias in which the dominant right side is generally longer [10]. This may cause an over- or underestimation of the pretraumatic length of an injured radius when using the opposite radius as guide.

Regarding the scaphoid, the level of bilateral symmetry has previously been investigated in several studies. Smith investigated left-to-right differences based on length, height, and intrascaphoid angle in 2D reconstructed sagittal and coronal sections from 30 healthy scaphoid pairs [20]. Heinzelmann et al. measured cadaveric scaphoids using a caliper [21]. In three 3D CT scaphoid studies, the length of long axes, volume, and surface area were measured [22–24]. In all aforementioned studies, on average, side-to-side differences were close to zero. However, these studies did not investigate symmetry of the relative position of the articular surfaces of scaphoid poles in terms of translational and rotational differences in 3D space. Three-dimensional information of these relative positions is of utmost importance in restoring normal alignment of the proximal and distal articular surfaces. It may help the surgeon in avoiding a scaphoid malunion in which the fragments have healed in malaligned configuration.

Although, on average, we found no left or right bias, in some individual cases, side-to-side differences were as large as 2 mm or 5–10°. These differences are small compared to values reported in clinical 3D CT studies investigating scaphoid nonunion deformity [2, 8, 25, 26]. Schweizer et al. found an average proximal translation of the distal pole of 3.3 mm and palmar flexion of 23° in 11 nonunions [2]. Thus, although perfect symmetry in individual cases was not observed, the contralateral side is still clinically useful as reference in reconstruction surgery.

The best reference is obviously the native, pretraumatic scaphoid itself, but scaphoid images before injury are only rarely available by coincidence. When using alternative references such as the contralateral side, one should question what difference between the postreduction and desired pretraumatic alignment can be considered clinically acceptable. This question is difficult to answer since recent articles focusing on the consequences of scaphoid malalignment are sparse. Some relatively old clinical articles suggested an association of malunion with pain, loss of motion and weakness after fracture healing [27–29] and with an increased risk of posttraumatic osteoarthritis [30]. In 1987, Burgess used four cadaveric wrists to simulate malunion [31]. He reported that an angular malalignment (i.e., palmar flexion of the distal pole) did not restrict wrist flexion and radial and ulnar deviation. Radiocarpal extension was reduced by 24° with 5° malalignment, and all extension was lost at 15° malalignment. In contrast, other studies concluded that there was no relationship of malunion with objective clinical outcome measures including range of motion and grip strength [6] or with long-term subjective outcome, including patient satisfaction [32]. All aforementioned studies, however, used 2D measures to assess malalignment including the intrascaphoid angle which are proven to be poorly reliable [3, 7]. Therefore, new biomechanical and clinical studies are needed to investigate the consequence of certain levels of malalignment, while using more reproducible measurement techniques, preferably in 3D space.

In conclusion, we proved that, on average, the articular surfaces of left and right scaphoid poles were symmetrically aligned. This suggests that the contralateral side is a useful reference in preoperative planning in reconstruction surgery of scaphoid fractures. Three-dimensional fracture displacement analysis provides objective information which may help the surgeon in characterizing complex fractures and surgical decision making.

## Figures and Tables

**Table 1:** Results of the left-to-right alignment differences of the distal poles represented by the six side-to-side differences based on the anatomical coordinate system. Negative displacement values represent opposite directions.

Displacement	Mean	Compared to 0 (*p* value)	SD
Translational			
Ulnar (mm)	0.1	0.50	0.6
Dorsal (mm)	0.4	0.11	1.2
Distal (mm)	0.2	0.17	0.6
Rotational			
Palmar rotation (deg.)	−1.1	0.32	4.9
Ulnar deviation (deg.)	−1.5	0.07	3.3
Pronation (deg.)	−1.0	0.27	3.7
